# Pembrolizumab-Induced Autoimmune Encephalitis: A Rare Case

**DOI:** 10.7759/cureus.102112

**Published:** 2026-01-22

**Authors:** Dalal A Obaid, Noora Jatan, Khawla Y Alawadhi, Minat Allah Alhusami, Khalid Haiba

**Affiliations:** 1 Medicine, Mohammed Bin Rashid University of Medicine and Health Sciences, Dubai, ARE; 2 Internal Medicine, Mohammed Bin Rashid University of Medicine and Health Sciences, Dubai, ARE; 3 Internal Medicine, Mohammed Bin Rashid University of Medicine and Health Sciences, dubai, ARE; 4 Critical Care Medicine, Mediclinic Parkview Hospital, Dubai, ARE

**Keywords:** autoimmune encephalitis, cancer immunotherapy, central nervous system toxicity, immune checkpoint inhibitors, immune related adverse events, neurological immune toxicity, pd-1 inhibitor, pembrolizumab

## Abstract

Immune checkpoint inhibitor-induced encephalitis (ICIiE) is a rare but potentially life-threatening neurological immune-related adverse event associated with cancer immunotherapy. Pembrolizumab, a programmed cell death protein 1 (PD-1) inhibitor, has demonstrated significant efficacy across multiple malignancies; however, its use can lead to dysregulated immune activation affecting the central nervous system. Clinical presentation is highly variable and often mimics infectious or paraneoplastic encephalitis, making diagnosis challenging, particularly in immunocompromised patients.

We report the case of a 63-year-old woman with grade III breast carcinoma, status post mastectomy, receiving adjuvant chemotherapy with paclitaxel and carboplatin in combination with pembrolizumab, who presented with persistent high-grade fever one week after immunotherapy administration. She was initially managed as a case of neutropenic sepsis and treated with broad-spectrum antibacterial and antifungal therapy; however, she developed progressive neurocognitive deterioration characterized by confusion, disorientation, irritability, and intermittent dystonic posturing, prompting intensive care unit admission. Extensive infectious evaluation, including repeated cultures, viral studies, and imaging, was unrevealing. Cerebrospinal fluid (CSF) analysis demonstrated marked lymphocytic pleocytosis and significantly elevated protein levels, while bacterial and viral polymerase chain reaction panels were negative. Brain MRI showed no definitive acute abnormalities. Given the temporal relationship with pembrolizumab exposure and exclusion of alternative etiologies, a diagnosis of immune checkpoint inhibitor (ICI)-related encephalitis was made. High-dose intravenous methylprednisolone was initiated, resulting in rapid defervescence and complete neurological recovery, normalization of inflammatory markers, and a return to baseline mentation.

This case highlights the importance of maintaining a high index of suspicion for immune checkpoint inhibitor-related encephalitis in patients receiving immunotherapy who present with unexplained fever and subacute neurological decline. Early multidisciplinary evaluation and prompt initiation of immunosuppressive therapy are critical to preventing morbidity and achieving favorable neurological outcomes.

## Introduction

Immune checkpoint inhibitors (ICIs) are monoclonal antibodies that have proven effective in treating various cancers, both early-stage and metastatic, by inhibiting pathways involved in maintaining immune tolerance and subsequently activating the immune system to destroy cancer cells de novo. Despite their numerous advantages, their side effect profile has been a major challenge, as they can adversely affect any system. Specifically, neurological side effects have been reported in up to 1% to 1.5% of patients treated with ICIs, but among them, ICI-induced encephalitis (ICIiE) is exceedingly rare, affecting just 0.1% to 0.2% of patients within weeks of ICI initiation [1,2]. It is most frequently encountered in patients with underlying lung cancer or melanoma, which represent the primary indications for ICI, with notable severity and mortality (up to 19% in some series) [3,4].

The clinical features of ICIiE are widely heterogeneous and can be grouped into two main syndromes: meningoencephalitis and focal encephalitis. Meningoencephalitis typically presents with a triad of fever, headache, and altered mental status. Focal encephalitis may manifest with symptoms and signs involving the limbic, diencephalic, brainstem, cerebellar, or other brain regions. Findings from cerebrospinal fluid (CSF) analysis are particularly helpful, as they are the most consistent, with nearly all patients showing lymphocytic pleocytosis, elevated protein, and normal glucose levels. In focal cases, oligoclonal bands may also be present. Serum antineuronal antibodies may be encountered in cases with a paraneoplastic component; otherwise, the majority of cases are seronegative. The MRI abnormalities are seen in about half the cases and may include T2/fluid-attenuated inversion recovery (FLAIR) hyperintensities in affected regions, meningeal enhancement, or patchy parenchymal lesions. Electroencephalogram readings may show a diffuse or focal slowing, but no pathognomonic pattern [4, 5].

Most patients with ICIiE respond favorably to high-dose intravenous corticosteroids, but since the differential diagnosis remains broad, empiric treatment for other causes, which may be life-threatening and treatable, must be initiated in parallel. When treated early, the prognosis for patients with ICIiE is overall favorable, with most patients experiencing recovery or significant improvement following immunosuppressive therapy. Markers of poor prognosis include the presence of focal syndromes, abnormal MRI findings, and antineuronal antibodies, whereas meningoencephalitic presentations with marked CSF inflammation and absence of autoantibodies are associated with a better prognosis [4]. Here, we present the case of a 63-year-old woman living in the United Arab Emirates who was treated in the intensive care unit for ICIiE one week following pembrolizumab therapy for breast cancer.

## Case presentation

A 63-year-old woman with a history of stage IIA breast carcinoma, pathological tumor size category T1c, pathological nodal involvement category N1, and no evidence of distant metastasis, status post mastectomy, with histopathology demonstrating invasive ductal carcinoma of the left breast with associated microcalcifications, and a triple-negative molecular profile (basal-like subtype), was receiving adjuvant chemotherapy with weekly paclitaxel and carboplatin and immunotherapy with pembrolizumab, an anti-programmed death-1 ICI, administered every three weeks. She presented with a persistent high-grade fever.

Her most recent chemotherapy cycle had been administered approximately one week prior to symptom onset, and subsequent treatment sessions were deferred following an episode of neutropenia. She was noted to be febrile during a routine oncology visit, with temperatures reaching 39.4°C. She reported chills, rigors, and night sweats but denied focal infective symptoms, including sore throat, dysuria, abdominal pain, diarrhea, cough, or pain and swelling at the portacath site. Despite repeated use of antipyretics, her fever persisted at or above 38°C. She was admitted under internal medicine with suspected neutropenic fever.

On admission, she was hemodynamically stable and saturating well on room air. Physical examination was largely unremarkable, with a soft, non-tender abdomen, clear lung fields, normal heart sounds, no focal neurological deficits, and a clean portacath site without local signs of infection. Laboratory investigations demonstrated an elevated C-reactive protein level of 149 mg/L and a mildly elevated procalcitonin level of 0.3 ng/mL. The initial neutropenia resolved, with recovery of the absolute neutrophil count to 3 × 10⁹/L. Serial blood, urine, and sputum cultures were negative, as were respiratory viral studies, including influenza and SARS-CoV-2. Chest radiography and abdominal ultrasound revealed no identifiable source of infection. Transthoracic echocardiography demonstrated preserved systolic function with grade I diastolic dysfunction and no evidence of valvular vegetations.

In the absence of a focal infective source, empirical broad-spectrum antimicrobial therapy was initiated with piperacillin-tazobactam and later escalated to meropenem. Piperacillin-tazobactam was administered for a total duration of five days, with the subsequent addition of linezolid and anidulafungin due to persistent pyrexia. A summary of peripheral blood laboratory investigations obtained on admission and at discharge is provided in Table 1.

**Table 1:** Peripheral blood laboratory investigations Peripheral blood laboratory parameters obtained on admission and at discharge demonstrate initial systemic inflammation with subsequent normalization following treatment.

Laboratory parameter	Reference range	On admission	At discharge
C-reactive protein (milligrams per liter)	0–5	146	4.8
Procalcitonin (nanograms per milliliter)	<0.05	0.3	0.1
Sodium (millimoles per liter)	136–145	143.3	141.1
Potassium (millimoles per liter)	3.5–5.1	3.82	3.53
Chloride (millimoles per liter)	98–107	103.3	103.8
Bicarbonate (millimoles per liter)	22–28	22.9	27
Magnesium (millimoles per liter)	0.66–0.99	0.978	1.04
White blood cell count (cells ×10³ per microliter)	4–11	3.2	11.4
Absolute neutrophil count (cells ×10³ per microliter)	1.9–8.2	1.63	10.03
Neutrophils (percentage)	Not applicable	50.57	87.7
Lymphocytes (percentage)	Not applicable	32.58	8.23
Monocytes (percentage)	Not applicable	15.78	3.94

Despite broad-spectrum antibacterial and antifungal therapy, the patient developed progressive neurological deterioration, characterized by acute irritability, disorientation, and confusion, prompting transfer to the intensive care unit. Her Glasgow Coma Scale (GCS) score declined to 14/15, with intermittent dystonic posturing of the right upper limb. There were no meningeal signs or focal neurological deficits. Given her clinical deterioration despite antimicrobial therapy, the differential diagnosis included persistent sepsis, infectious meningoencephalitis, paraneoplastic limbic encephalitis, and immune-related encephalitis secondary to pembrolizumab. After a multidisciplinary discussion involving neurology and oncology, a lumbar puncture was performed. 

**Figure 1:**
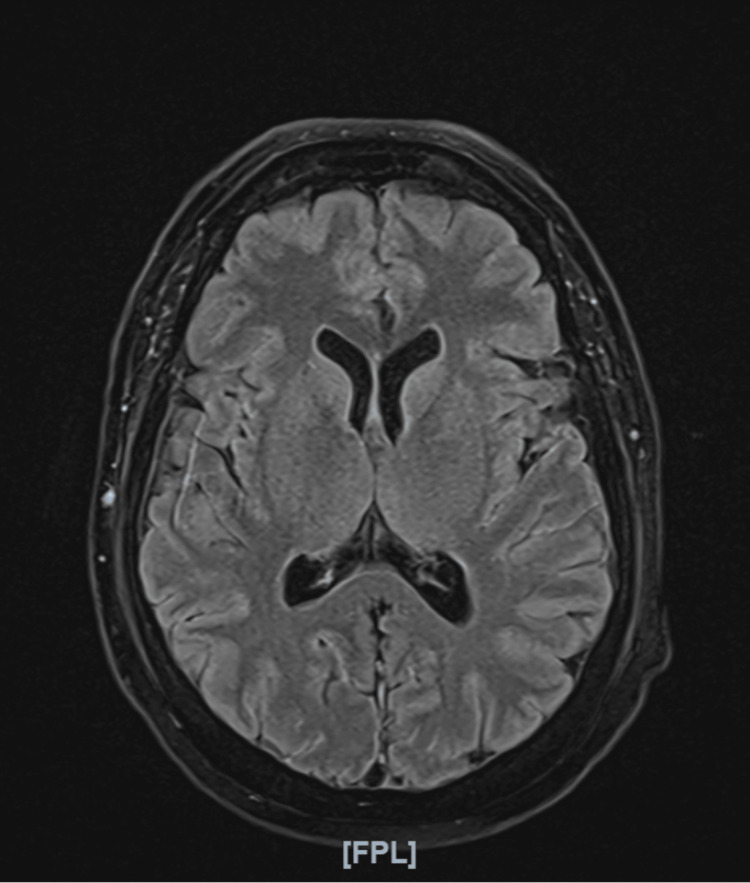
Axial T2 FLAIR MRI of the brain showing no acute intracranial abnormalities FLAIR: Fluid-attenuated inversion recovery

**Figure 2:**
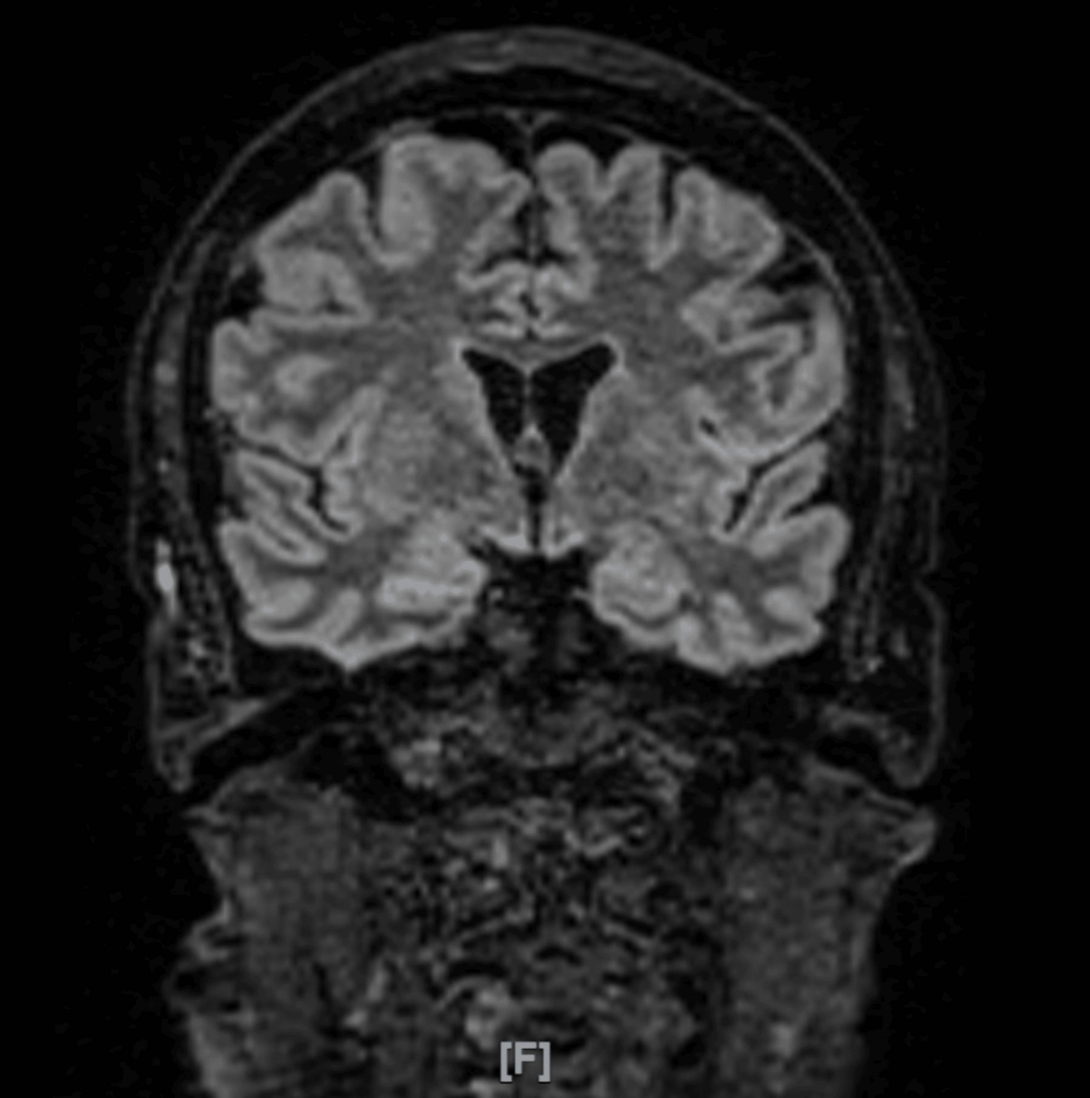
Coronal T2 FLAIR MRI of the brain showing no acute intracranial abnormalities FLAIR: Fluid-attenuated inversion recovery

The CT-guided lumbar puncture yielded clear CSF with marked lymphocytic pleocytosis and significantly elevated protein levels exceeding 1500 mg/l. Gram stain, bacterial cultures, and multiplex polymerase chain reaction testing for bacterial and viral meningitis, including herpes simplex virus types 1 and 2, were negative. A brain MRI was obtained to evaluate for encephalitic changes. The brain MRI did not demonstrate any acute intracranial abnormalities or radiological features suggestive of encephalitis (Figures 1-2). The CSF laboratory findings are summarized in Table 2.

**Table 2:** CSF laboratory investigations obtained during hospitalization Lab results showed lymphocytic pleocytosis and markedly elevated protein levels in the absence of identified infectious pathogens. CSF: Cerebrospinal fluid

Laboratory parameter	Reference range	Finding
Glucose (millimoles per liter)	2.22-3.88	3.76
Protein (milligrams per liter)	150-450	1502
Red blood cells (cells per cubic millimeter)	0-2	16
White blood cells (cells per cubic millimeter)	0-5	89
Neutrophils (percentage)	0-6	2
Lymphocytes (percentage)	40-80	77
Monocytes (percentage)	15-45	21

Extensive antibody testing for autoimmune, paraneoplastic, and infectious disorders was performed as part of the diagnostic evaluation. Autoimmune encephalitis antibodies tested included antibodies against the N-methyl-D-aspartate receptor, antibodies against the alpha-amino-3-hydroxy-5-methyl-4-isoxazolepropionic acid receptor subtypes 1 and 2, antibodies against the gamma-aminobutyric acid B receptor, antibodies against leucine-rich glioma-inactivated protein 1, antibodies against contactin-associated protein-like 2, antibodies against dipeptidyl-peptidase-like protein 6, amphiphysin antibodies, collapsin response mediator protein 5 antibodies (CV2), paraneoplastic antigen Ma2 antibodies, anti-neuronal nuclear antibody type 1 (Hu), anti-neuronal nuclear antibody type 2 (Ri), Purkinje cell cytoplasmic antibody type 1 (Yo), recoverin antibodies, SRY-box transcription factor 1 antibodies, titin antibodies, and zinc finger protein 4 antibodies, all of which were negative. Additional testing for viral, bacterial, fungal, and rheumatologic etiologies was negative.

In the setting of persistent fever, repeatedly negative infectious investigations, and progressive neurocognitive decline, the multidisciplinary team comprising oncology, neurology, infectious diseases, and intensive care specialists favored a working diagnosis of ICI-related encephalitis. Given the lack of clinical response to broad-spectrum antimicrobial therapy for presumed sepsis, checkpoint inhibitor-related encephalitis was considered the leading diagnosis.

High-dose intravenous methylprednisolone (1 g daily for five days) was initiated after approximately one week of ongoing antimicrobial therapy, followed by a tapering regimen of oral prednisolone starting at 60 mg daily with stepwise dose reduction. The patient demonstrated rapid clinical improvement, with defervescence, recovery of orientation, and return to baseline mentation, achieving a GCS score of 15/15. Inflammatory markers declined, with C-reactive protein decreasing to 11 mg/L, and no infectious source was identified during hospitalization.

The patient was subsequently transferred from the intensive care unit to the medical ward in a fully alert and oriented state, tolerating oral intake and ambulating with support. Discharge planning included a supervised steroid taper and close outpatient follow-up with oncology and neurology. At follow-up, the patient remained neurologically stable with sustained clinical recovery and no recurrence of neurocognitive symptoms following completion of the steroid taper.

## Discussion

Pathophysiology

Immune checkpoint inhibitor-induced encephalitis is a rare but serious immune-related adverse event caused by uncontrolled immune activation following inhibition of regulatory immune pathways, including cytotoxic T-lymphocyte-associated antigen 4 (CTLA-4), programmed cell death protein 1 (PD-1), and programmed death ligand 1 (PD-L1). Blockade of these inhibitory pathways enhances T-cell-mediated immune responses against tumor cells but may also promote autoreactivity against self-antigens within the central nervous system (CNS), resulting in neuroinflammation and neuronal injury [6]. The pathophysiology is multifactorial and involves several interconnected immune mechanisms.

T-cell-mediated autoimmunity is considered the primary pathogenic mechanism. Histopathological and immunological studies have demonstrated infiltration of the CNS by activated CD4⁺ and CD8⁺ T lymphocytes, often accompanied by oligoclonal T-cell expansion and cytotoxic neuronal damage [6]. In addition, immune checkpoint inhibition disrupts immune tolerance by impairing both peripheral and central regulatory mechanisms that normally prevent autoreactive lymphocytes from targeting self-antigens. This loss of tolerance can lead to de novo autoimmune encephalitis or exacerbate preexisting paraneoplastic neurological syndromes [7].

A subset of patients develops autoantibodies directed against neuronal antigens, including cell-surface antigens such as N-methyl-D-aspartate receptor (NMDAR) or intracellular onconeural antigens such as Hu and Ma2. These autoantibodies may arise de novo or be unmasked following ICI therapy and are often associated with paraneoplastic neurological syndromes and less favorable clinical outcomes [8]. Furthermore, a pro-inflammatory cytokine milieu, characterized by elevated cytokines and chemokines in both CSF and serum, amplifies neuroinflammation and accelerates neuronal injury [9].

Diagnostic dilemmas

Diagnosing ICIiE is challenging due to its heterogeneous clinical presentation and the absence of pathognomonic biomarkers or imaging findings. Patients commonly present with subacute neurological symptoms, including altered mental status, confusion, seizures, or focal neurological deficits, which overlap with infectious, paraneoplastic, and other autoimmune encephalitides, such as herpes simplex virus encephalitis or anti-leucine-rich glioma-inactivated 1 (LGI1) autoimmune encephalitis [10].

Most patients with ICIiE have negative antineuronal antibody panels and nonspecific MRI findings. The CSF analysis typically reveals lymphocytic pleocytosis and elevated protein levels; however, these findings are not unique to immune-mediated encephalitis and may also be seen in infectious or paraneoplastic processes. In some cases, ICI therapy may unmask an underlying paraneoplastic neurological syndrome, which is associated with a worse prognosis and is clinically indistinguishable from classic paraneoplastic encephalitis apart from its temporal association with immunotherapy [4]. The diagnostic process is further complicated by the need to exclude infectious etiologies, metastatic disease, metabolic disturbances, and other medication-related neurotoxicities.

Immune checkpoint inhibitor-induced encephalitis remains primarily a clinical diagnosis established in the context of recent ICI exposure, subacute neurological deterioration, and supportive paraclinical findings after exclusion of alternative diagnoses. Proposed diagnostic criteria include symptom onset within three months of ICI initiation, altered mental status or cognitive decline, psychiatric symptoms, or working memory deficits, in addition to at least one supportive finding such as new focal CNS signs, unexplained seizures, CSF pleocytosis (>5 white blood cells/µL), or MRI changes suggestive of encephalitis [11].

Treatment and response

High-dose corticosteroids are the cornerstone of treatment for ICIiE. In reported cases, nearly all patients received intravenous corticosteroids, most commonly methylprednisolone at doses of 500 mg to 1000 mg daily for three to five days, followed by a gradual oral taper [5,12]. The majority of patients demonstrate rapid and significant clinical improvement after steroid initiation. Although relapse after steroid tapering has been reported, it remains uncommon and underscores the importance of a slow and carefully monitored tapering regimen [12].

Early recognition and prompt initiation of immunosuppressive therapy are strongly associated with favorable neurological outcomes. Müller-Jensen et al. demonstrated that early immunosuppression correlated with complete neurological recovery in affected patients, and multiple case reports emphasize that very high initial steroid doses may yield rapid clinical benefit [10,12]. In cases of severe disease or incomplete response to corticosteroids, additional immunomodulatory therapies may be required. According to Stuby et al., steroid unresponsiveness is rare and should prompt reconsideration of the diagnosis [5]. Second-line therapies include intravenous immunoglobulin, plasmapheresis, or rituximab [1,5].

Current recommendations uniformly advise withholding ICI therapy, initiating urgent high-dose corticosteroids, and escalating to additional immunotherapy if clinical improvement is insufficient. With this approach, mortality remains low, and neurological recovery is often complete [5,10].

Comparison with existing literature 

The clinical course of our patient is consistent with features described in recent case series and systematic reviews. Immune checkpoint inhibitor-induced encephalitis typically occurs subacutely following immunotherapy initiation. Reported median latency is approximately 65 days, although onset may range from days to more than one year after exposure [5,10]. Common presenting symptoms include altered mental status, confusion, decreased consciousness, seizures, and focal neurological deficits [5]. The MRI findings are frequently normal or show nonspecific abnormalities such as T2-weighted white matter hyperintensities or contrast enhancement [10]. The CSF analysis usually demonstrates lymphocytic pleocytosis and elevated protein levels, while infectious and neoplastic causes must be rigorously excluded [5]. Electroencephalogram abnormalities, including diffuse slowing or epileptiform discharges, are nonspecific and occur in a minority of cases [10].

Velasco et al. identified two principal clinical phenotypes: focal encephalitis and diffuse meningoencephalitis, with outcomes influenced by antibody status and clinical presentation. Patients without detectable neuronal autoantibodies generally exhibit favorable steroid responsiveness, whereas those with paraneoplastic-type antibodies tend to have poorer outcomes. Undetected preexisting paraneoplastic encephalitic syndromes have been identified as a risk factor for unfavorable prognosis [4]. These findings support the concept that ICI therapy may unmask latent autoimmune CNS disorders or induce a steroid-responsive inflammatory encephalitis.

Although most reported cases have occurred in patients with melanoma or lung cancer, encephalitis has been documented across tumor types and ICIs. A recent case described pembrolizumab-induced encephalitis in a patient with triple-negative breast cancer presenting with expressive aphasia just three days after treatment initiation [13]. These observations emphasize that neurological immune-related adverse events may occur even in newer immunotherapy indications, highlighting the need for vigilance across all oncology specialties.

Future directions

Immune checkpoint inhibitor-induced encephalitis remains a poorly defined entity, and significant gaps in knowledge persist. No specific diagnostic biomarkers have been identified, as routine autoantibody panels are frequently negative. In Müller-Jensen’s cohort, only one of 30 patients demonstrated a known neuronal autoantibody, and antibody positivity rates across studies range from approximately 37% to 58% [10]. The MRI and electroencephalogram findings lack specificity, and routine laboratory markers are often nonspecific. These limitations underscore the need for further research into novel diagnostic tools, including biomarkers such as neurofilament light chain, glial fibrillary acidic protein, and cytokine profiling.

Several clinical questions remain unanswered, particularly regarding ICI rechallenge. Buckley et al. reported that only 5% of patients were rechallenged, with nearly half experiencing recurrent neurological symptoms, suggesting that rechallenge should be considered only in exceptional circumstances following careful risk-benefit assessment [1]. Long-term neurological outcomes also remain incompletely characterized. Although short-term recovery is common, persistent cognitive or functional deficits have been reported among survivors, highlighting the importance of long-term follow-up and rehabilitation [1].

Finally, improved diagnostic frameworks and standardized guidelines are needed. Current immune-related adverse event protocols emphasize prompt steroid initiation and exclusion of mimics, but formal diagnostic criteria analogous to those used for autoimmune encephalitis may facilitate earlier recognition. Given the frequent coexistence of other immune-related adverse events, clinicians should maintain a high index of suspicion for neurological toxicity in any patient receiving ICI therapy [10]. Multicenter collaboration and prospective registries will be essential to improve diagnostic accuracy, management strategies, and long-term outcome assessment.

## Conclusions

This case illustrates ICIiE, a rare but serious neurological immune-related adverse event that can closely mimic other etiologies of encephalitis. Our patient developed acute confusion and fever one week after pembrolizumab therapy. A comprehensive infectious disease evaluation yielded negative results, while CSF analysis confirmed immune-mediated neuroinflammation. Early recognition and prompt initiation of high-dose intravenous corticosteroids resulted in rapid and complete neurological recovery. This case underscores the importance of maintaining a high index of suspicion for ICI-related neurotoxicity in patients presenting with new neurological symptoms following immunotherapy. As the use of ICIs continues to expand, increased awareness of their potential neurological adverse events and a multidisciplinary approach are essential to optimize patient outcomes.
